# The Skin

**Published:** 1914-04-11

**Authors:** J. H. Sequeira

**Affiliations:** Physician to the Skin Department, London Hospital


### COMMON ERRORS IN DIAGNOSIS.

### The Skin.

By Dr. J. H. SEQUEIRA, M.D., F.R.C.P., F.R.C.S., Physician to the Skin Department, London Hospital.

(Report in abstract of a Lecture delivered at the Polyclinic.)

The diagnosis of skin diseases is often difficult. The examining bodies do not take much interest in dermatology, and the senior student can rarely find time for work in a skin clinique.

The skin disease most frequently missed in private practice is scabies, largely because, in cleanly persons, the eruption is not suspected, and often lacks some of the common features. The disease should be thought of whenever a person complains of intense itching of the skin, particularly at night. The chief point to remember is its centripetal character; it spreads from the extremities to the trunk. One should examine first the inter-digital clefts, then the inner side of the wrists, the anterior fold of the axilla, and, in the male, the external genitals. The diagnostic feature is the burrow, a brownish linear elevation of the skin, about a quarter of an inch in length. This burrow should be opened with a needle, and the acarus sought. "Itch" is frequently mistaken for impetigo, and sometimes for eczema. Attention was directed to sulphur dermatitis following scabies, a condition often leading to persistence in the use of the drug—under the impression that the scabies was not cured.

### Drug Eruptions.

With regard to drug eruptions, some are caused by local application of remedies to the skin, others result from the internal administration of certain drugs. On some people boric acid causes an eczematous dermatitis starting from some trivial lesion; and most antiseptic applications act as irritants to certain skins. In one case of an eruption the patient had been using capsicum wool as a prophylactic against bronchitis. Atropine dermatitis is sometimes overlooked; it is usually caused by the instillation of drops of atropine into the eye; sometimes a lotion of that drug has been applied.

The eruptions caused by the internal administration of drugs are more difficult to recognise, and in the absence of a history even experienced dermatologists may fail to recognise it. In some, quinine causes a scarlatiniform eruption, followed by desquamation. Salicylic compounds may produce erythema, as may also belladonna. Copaiba eruptions often lead to error; they have been mistaken for measles; secondary syphilides for urticaria. The copaiba eruption is widely spread, first appearing on the front of the thighs; the macular syphilide is never so diffuse and does not itch, while the drug eruption usually irritates. In cases of doubt, the Wassermann test should be made. Bromide eruptions are frequently overlooked, though the acne in patients suffering from epilepsy is very important. Many infants show an idiosyncrasy for bromide of potassium, which produces in them a peculiar soft warty eruption, which is often mistaken. With regard to iodide eruptions, there are several forms, the most important of which begins with an outbreak of small lesions looking like vesicles. These may ulcerate, rupture, and form crusts. In other cases there may be blisters or bullæ containing serous fluid. They early become filled with what looks like granulation tissue. The condition has been diagnosed as mycosis fungoides, and as tertiary syphilis, and with such there is risk in giving heavy doses of the drug; it should be stopped, and free elimination encouraged. The death of a patient suffering from cardiac disease may be hastened by the administration of this drug. Purpura is a rare effect of giving iodides.

### Eruptions Mistaken for Syphilis.

With reference to acute specific fevers, the experienced family practitioner is not likely to overlook an acute exanthem and consider it a cutaneous disease, but measles in a young adult has been diagnosed as urticaria. Acute erythematous eczema of the face is frequently called erysipelas, but there is no pyrexia, and the general symptoms of erysipelas are lacking. In such a case it is very important to take the temperature.

An important part of the subject concerns eruptions which are commonly mistaken for syphilis. The late Sir Jonathan Hutchinson rightly said that pityriasis rosea was more often considered a secondary syphilide than anything else was. The eruption of pityriasis rosea consists of lozenge-shaped or rounded pink, scaly spots; it is generally confined to the trunk and adjacent parts of the extremities; often it has a "vest" distribution. Itching is variable, and rarely severe. Usually the patient with pityriasis rosea is able to say that one spot preceded most of the eruption by a few days, or even weeks; and this "herald spot" is generally large and distinctive. The macular syphilide is the earliest general eruption, and the spots are of equal size, pink and not scaly, and they do not itch, neither is there any infiltration. In pityriasis rosea there are no general enlargements of the glands, and the Wassermann test is negative. The scaly syphilide must be distinguished from pityriasis rosea; the former is an eruption of papules on which scales develop, and the papule is generally of coppery colour. In pityriasis rosea the eruption is all scaly. The great safeguard is the Wassermann test. Lichen planus is often diagnosed as syphilis, but lichen planus has a lavender or violet colour, and it consists of small, flat-topped shiny papules. In about half the cases of lichen planus the lesions occur on the buccal mucous membrane, and this fact often makes the practitioner think of syphilis. The acute eruption always itches, in which respect it differs from the secondary syphilide.

With regard to the diagnosis between lupus and syphilis, the rate of destruction of tissue which has ensued is a strong point of difference. Dr. Payne's comparison, that the march of lupus was like the hour hand of a clock, while that of syphilis was like the minute hand's progress, was instructive and true. Again, extensive destruction of bone does not occur in tuberculosis, only in connection with syphilis. In the later stages of congenital syphilis there may be a difficulty in distinguishing it from lupus, but here again rapidity of development is important. Intranasal lupus often starts with epiphora, and it is not until the skin is affected that lupus is suspected. Epiphora must be taken as an early sign of intranasal lupus.

Ringworm of the scalp is frequently overlooked. In the microsporon forms there is often a scaly eruption which may be mistaken for dandruff or scurf. The broken hair should be examined. One type of endothrix ringworm produces a bald patch; in another the skin is bald and studded over with black dots, which represent the hairs broken off flush with the scalp. These conditions are often thought to be alopecia areata. A form occurs in girls in which the hair breaks off at varying lengths. The fungus lies in the hair and is not easily demonstrated. An eczematoid ringworm in the groin is somewhat common in men; it is a red patch with scales, and a festooned margin, situated on the inner part of the thigh, and extending to the adjacent part of the scrotum. With this is often associated an eczema between the toes; the cause in both conditions is a special ringworm fungus, the *Epidermophyton inguinale*.

Lastly, one should have in mind the possibility that an unusual eruption has been produced by the patient himself, with the object, usually, of exciting sympathy, or as an excuse for laziness in the case of a workman. Malingering is nowadays quite common, and in many cases the man receives more money when he is not working than when he is. The fact that an eruption has a peculiar shape should be noted; these artefact lesions usually have straight lines, which natural lesions have not.

